# Individual differences in working memory capacity and workload capacity

**DOI:** 10.3389/fpsyg.2014.01465

**Published:** 2014-12-18

**Authors:** Ju-Chi Yu, Ting-Yun Chang, Cheng-Ta Yang

**Affiliations:** Department of Psychology, National Cheng Kung UniversityTainan, Taiwan

**Keywords:** executive function, linear ballistic accumulator model, systems factorial technology, working memory capacity, workload capacity

## Abstract

We investigated the relationship between working memory capacity (WMC) and workload capacity (WLC). Each participant performed an operation span (OSPAN) task to measure his/her WMC and three redundant-target detection tasks to measure his/her WLC. WLC was computed non-parametrically (Experiments 1 and 2) and parametrically (Experiment 2). Both levels of analyses showed that participants high in WMC had larger WLC than those low in WMC only when redundant information came from visual and auditory modalities, suggesting that high-WMC participants had superior processing capacity in dealing with redundant visual and auditory information. This difference was eliminated when multiple processes required processing for only a single working memory subsystem in a color-shape detection task and a double-dot detection task. These results highlighted the role of executive control in integrating and binding information from the two working memory subsystems for perceptual decision making.

## Introduction

The present study aimed to investigate the relationship between two capacity measures: working memory capacity (WMC) in the literature of working memory (Baddeley and Hitch, 1974; Barrett et al., 2004) and workload capacity (WLC) in the literature of perceptual decision making (Townsend and Ashby, 1978; Townsend and Nozawa, 1995; Townsend and Eidels, 2011). Although both measures assess an individual's information processing capacity, it was unclear whether the two capacity measures assess a unitary, central capacity of an information processing system. We used a non-parametric approach (systems factorial technology, SFT) (Townsend and Nozawa, 1995) and a parametric approach (linear ballistic accumulator model, LBA) (Brown and Heathcote, 2008; Eidels et al., 2010) to assess WLC in different task contexts and examined individual differences in WLC and WMC. We will briefly introduce the concepts of the two capacity measures.

Working memory refers to aspects of on-line cognition, such as monitoring, processing, and maintenance of information. A key component of Baddeley and Hitch's (1974) model of working memory, also known as the “short-term storage” of information (Henderson, 2013), is the central executive system, which is a modality-free function that supervises two slave systems of working memory: the phonological loop and the visuospatial sketchpad. The central executive system plays an important role in integrating information from the two subsystems for manipulation and operation. Following Baddeley and Hitch (1974), many theories regarding the construct of the central executive system have been proposed—for example, the *supervisory attention system* (SAS) in Norman and Shallice (1986) and the *executive control* in Posner and Digirolamo (2000). WMC is an index that denotes the capability of attention control in central executive of a working memory system and researchers typically use a counting span task (Case et al., 1982), an operation span task (OSPAN task) (Turner and Engle, 1989), and a reading span task (Daneman and Carpenter, 1980) to measure one's WMC. Measures of WMC are strongly related to general fluid intelligence (Conway et al., 2003) and show considerable construct validity insofar as they predict performance on a wide range of tasks that require domain-general controlled attention. WMC is different from the traditional concept of short-term memory capacity, which is thought to reflect primarily domain-specific storage. One of the most widely supported theories, particularly when applied to individual differences in working memory, is the attention control theory of working memory (Engle and Kane, 2004). Individuals with high WMC have greater attention control in integrating information from different domain-specific subsystems (Rosen and Engle, 1997; Engle et al., 1999; Barrett et al., 2004; Engle and Kane, 2004). These results have been supported by computational modeling research (Anderson, 2013) and neurobiological research (Miller and Cohen, 2001).

At approximately the same time, another capacity measure, WLC was developed (Townsend and Ashby, 1978; Wenger and Gibson, 2004; Townsend and Eidels, 2011). WLC is also known as perceptual capacity. In contrast to WMC, which measures an individual's capacity to maintain and process information, WLC measures the efficiency of perceptual processing as workload (i.e., the number of channels or signals to be processed) increases. If the processing rate of an individual channel does not change as the workload increases, the system is described as unlimited-capacity processing. If the individual-channel processing speed slows down with an increasing workload, the system is described as limited-capacity processing, and if processing speeds up, the system is described as supercapacity processing. WLC is commonly measured with a redundant-target detection task (Miller, 1982; Townsend and Nozawa, 1995) where participants are required to monitor two sources of information. Participants have to make a positive response when they detect the presence of both of the targets (redundant-target condition) or either target (single-target condition); otherwise, they have to make a negative response when they detect neither target (no-target condition). WLC can be assessed by comparing the reaction time distributions between the redundant-target and single-target conditions. For more theoretical derivations, please see Townsend and Nozawa (1995) and Wenger and Gibson (2004). Previous studies have widely applied the measure of WLC to study how people process multiple sources of information and how this measure is related to different aspects of human cognition. For example, in a double-dot detection task, participants were of limited-capacity in processing redundant spatially-independent visual information (Townsend and Nozawa, 1995; Eidels et al., 2010), which was against the prediction from the unlimited-capacity, independent, parallel (UCIP) model. In a redundant color-shape detection task, participants were of unlimited-capacity in processing separable perceptual dimensions when inter-stimulus contingency information was removed (Mordkoff and Yantis, 1991, 1993). In a visual search task, participants were of supercapcity in searching for a feature singleton defined by luminance and/or orientation (Zehetleitner et al., 2009). In a visual-auditory detection task, participants were of supercapacity in processing multisensory information (Miller, 1982), which was known as an effect of “multisensory integration” (Hugenschmidt et al., 2010; Altieri and Townsend, 2011).

In addition to WLC, there are two other important characteristics to describe information processing in a system, including the processing architecture (serial vs. parallel vs. coactive) that denotes the order of multiple-signal processing and the decisional stopping rule (self-terminating vs. exhaustive) that denotes the amount of information required for a decision. Although WLC and the processing architecture are two independent measures of information processing (Townsend and Nozawa, 1995), WLC may constrain the order of multiple-signal processing. For example, a standard serial model is assumed to involve limited-capacity processing (Townsend and Ashby, 1983); an independent parallel model usually involves unlimited-capacity processing, which is known as the UCIP (Houpt and Townsend, 2012); and a coactive model is assumed to involve supercapacity processing (Wenger and Townsend, 2001). On the other hand, a recent simulation study (Eidels et al., 2011) demonstrated that a parallel model with supercapacity processing suggests the existence of facilitatory between-channel crosstalk during the stage of information accumulation, whereas a parallel model with limited-capacity processing suggests an inhibitory interaction between channels.

Both WMC and WLC represent a system's capacity to process information, but they are different constructs in nature. The processing capacity in a working memory system describes the capacity of domain-general controlled attention to maintain and process information and, especially, integrate information from the two subsystems. In contrast, WLC represents a system's capacity of multiple-signal processing and is referred to as the variation of the processing efficiency of an individual channel as a function of workload. The relationship between WMC and WLC remains unclear, however, and to our knowledge, no prior studies have investigated the relationship between the two constructs, except for a recent study conducted by Heathcote et al. (2014).

The present study examined the relationship between WMC and WLC. To measure WMC, participants were asked to performed an OSPAN task, in which they had to remember a few words while solving an arithmetic equation at the same time (Turner and Engle, 1989). In addition, they performed three different redundant-target detection tasks to measure their WLC. Modalities that the participants had to supervise in three redundant-target detection tasks were well defined; redundant information may come from a single visual modality (two visual features, two distinct spatial positions) or two different modalities (i.e., visual and auditory modalities). The reasons why we chose these tasks were as follows: (1) These redundant-target detection tasks have been widely used to study multiple-signal processing in the previous literature (Miller, 1982; Mullin et al., 1988; Townsend and Nozawa, 1995; Eidels et al., 2010), but less is known about the individual variation of the perceptual processing capacity in different tasks. (2) Relating WLC and WMC in different task contexts enables us to examine whether it requires a unitary, central capacity of information processing to process multiple signals that come from the same or different modalities. If both WMC and WLC assess the central processing capacity, we expect WLC to be positively related to WMC, regardless of whether redundant information is from the same modality. These results can shed light on the nature of the working memory system and the role of executive control in processing multiple signals for perceptual decision making.

## Experiment 1

In Experiment 1, an OSPAN task was conducted to measure the participants' WMC and three redundant-target detection tasks— i.e., a color-shape detection task, a double-dot detection task, and a visual-auditory detection task— were conducted to measure their WLC. We expect that participants high in WMC would have larger WLC in multiple-signal processing.

### Method

#### Participants

Fifty-seven (29 males and 28 females) undergraduates with a mean age of 20.63 years (*SD* = 2.72) at National Cheng Kung University volunteered in this experiment. All the participants had normal or corrected-to-normal vision and hearing. They signed a written informed consent prior to the experiment and received NTD 120 per hour after they completed the experiment.

#### Equipment

All the stimuli were presented on a 19-inch CRT monitor (CTX) with a refresh rate of 85 Hz and a display resolution of 1024 × 768 pixels. The viewing distance was 60 cm. Auditory stimuli were presented via a Philips Shm6500 headphone. The experiment was programmed with E-prime 1.1 (Schneider et al., 2012).

#### Stimuli, design, and procedure

Each participant performed three redundant-target detection tasks to measure his/her WLC and an OSPAN task to measure the capacity of a dynamic working memory system that involved both the storage and processing of information. Each task last for approximate 1 hour and four tasks were conducted on different days.

In the color-shape detection task, a test display consisted of a letter that was either an O or an X in shape, either green or cyan in color, and 1° (horizontal) × 1° (vertical) in size. The target color was defined as green, and the target shape was defined as X. In the redundant-target condition, the test stimuli consisted of both the target color and target shape (green X). In the single-target condition, the test stimuli consisted of either the target color or target shape (green O, cyan X). In the no-target condition, the test stimuli consisted of neither the target color nor target shape (cyan O). Each condition was equally probable and randomly intermixed within a block. After the participants practiced for 40 trials, they performed 12 blocks of 80 test trials.

**Figure 1:**
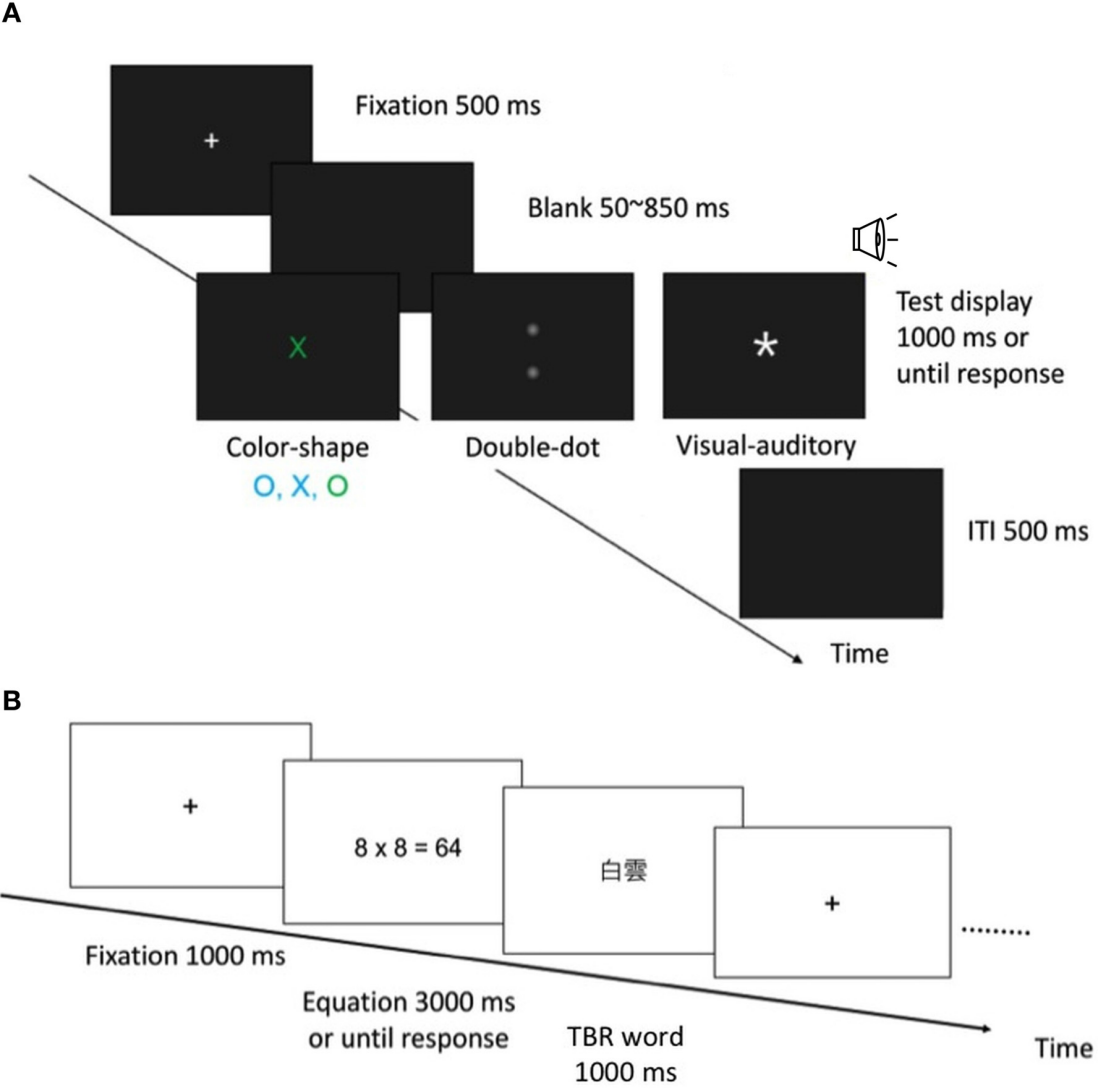
**(A)** An illustration of the experimental procedure in the color-shape, double-dot, and visual-auditory detection tasks. **(B)** An illustration of the experimental procedure in the OSPAN task.

Each trial began with a 500 ms fixation point (see Figure 1A for an illustration). Following a uniformly distributed random foreperiod ranging from 50 to 850 ms, a test stimulus was presented until participants responded or 1000 ms elapsed. Participants had to make a go/no-go response as quickly as possible when they detected either target feature (green or X). If *either* or *both* target features were detected, participants were required to press the “/” button (go response); if *neither* target feature was detected, they had to hold their response and wait for the next trial (no-go response). The inter-trial interval (ITI) was 500 ms.

In the double-dot detection task, the design and procedure were the same as those used in the color-shape detection task, except for the test stimuli. A 1° × 1° light dot (*luminance* = 0.031 *cd/m*^2^) was presented 6° above and/or below the fixation point^1^. There were three types of test trials: redundant-target condition (both locations contained a light dot), single-target condition (either the top or bottom location contained a light dot), and no-target condition (neither location contained a light dot). Participants had to detect the presence of either or both dots as quickly as possible; otherwise, they had to hold their response and wait for the next trial (see Figure 1A).

In the visual-auditory detection task, the design and procedure were also the same as the former two tasks, except for the test stimuli, which consisted of a star sign (1° × 1°, *luminance* = 29.4 *cd/m*^2^) and/or a 750-Hz pure tone (47.5 db). There were three types of test trials: redundant-target condition (both visual and auditory signals were presented), single-target condition (either visual or auditory signal was presented), and no-target condition (neither visual nor auditory signal was presented). Participants had to detect the presence of either or both the visual and auditory targets as quickly as possible; otherwise, they had to hold their response (see Figure 1A).

In the OSPAN task, participants first saw an arithmetic equation, for example, 8 × 8 = 64, then they had to indicate whether the presented answer was correct, and finally saw a to-be-remembered (TBR) two-character Chinese word for later recall (see Figure 1B for an illustration). In each trial, there were two to six such processing-and-storage presentations. After the presentations, participants were required to write down the TBR words in correct serial order. There were a total of 15 trials that consisted of 5 presentation conditions (2/3/4/5/6) and three trials per condition. All the trials were randomly presented.

#### Data analysis

Reaction time data of the correct responses in the redundant-target detection tasks was analyzed to estimate WLC. According to SFT (Townsend and Nozawa, 1995; Townsend and Eidels, 2011), the capacity coefficient is expressed as follows:

(1) $$C\left(t\right)=\frac{\log\left[S_{1,2}\left(t\right)\right]}{\log\left[S_{1}\left(t\right)\times S_{2}\left(t\right)\right]}$$

for *t* > 0, *S*_1_*(t)*, *S*_2_(*t*), and *S*_1,2_(*t*) represent the survivor function, the complement of the cumulative probability function [1-*F(t)*], of the two single-target conditions and a redundant-target condition, respectively. The capacity coefficient provides a comparison of the amount of work that is completed by the system while processing redundant targets and the summed amount of work that is completed by each single target processed individually at the same amount of time. A value of *C(t)* = 1 suggests unlimited-capacity processing: the processing efficiency of an individual channel is *not* affected by the change in workload. *C(t)* > 1 suggests supercapacity processing: increasing the to-be-processed signals speeds up the processing time of an individual channel. *C(t)* < 1 indicates limited-capacity processing: increasing the workload slows down the processing time of an individual channel.

To assess WMC for each participant, we first computed the recall score for each trial, which was defined as the number of TBR words fully recalled in correct serial order. WMC was computed by summing the recall scores of all the trials. The recall score ranges from 0 to 60.

#### Result

The number of correct answers on the processing component of the OSPAN task (i.e., solving the arithmetic equation) was analyzed. Four participants' data were excluded from further analysis because their processing accuracy was below 0.7. Under this criterion, the mean processing accuracy was 0.85 with a standard deviation of 0.06. We then computed the total number of items recalled from the storage component of the OSPAN task (i.e., recall score). The mean recall score was 36.38 with a standard deviation of 10.49.

We then conducted an extreme-group approach to investigate the relationship between WMC and WLC. This approach has been widely used to analyze continuous variables (Preacher et al., 2005). We selected the subject for further analysis on the basis of the extreme WMC scores (i.e., recall scores in the OSPAN task) to emphasize the differences in WLC between the high-WMC and low-WMC groups. The high-WMC group included the participants with the top 30% of recall scores (*M* = 47.33, *SD* = 4.45, *N* = 18), and the low-WMC group included the participants with the bottom 30% of recall scores (*M* = 24.44, *SD* = 5.49, *N* = 18). The recall scores of the two groups were significantly different [*t*_(34)_ = 13.75, *p* < 0.0001].

To do further analysis, we then excluded the trials with reaction time less than 150 ms in the redundant-target detection tasks. This criterion was selected because simple reaction times are generally slower than 150 ms. The mean performance of the redundant-target detection tasks for each group was summarized in Table 1. Accuracies were very high across conditions for both groups of participants except for the performance in the no-target condition of the color-shape detection task (0.89), suggesting a potential response bias in detecting color and/or shape. A Two-Way (high-WMC/low-WMC group × redundant-target/single-target condition) analysis of variance (ANOVA) was conducted to analyze the accuracy and correct reaction time data of the three tasks. We found that all the effects were not significant for the accuracy data of all the tasks. For reaction time data, there were significant main effects of group [CS^2^: *F*_(1, 68)_ = 9.00, *p* < 0.005; DD: *F*_(1, 68)_ = 7.27, *p* < 0.01; VA: *F*_(1, 68)_ = 13.31, *p* < 0.001] and condition [CS: *F*_(1, 68)_ = 33.50, *p* < 0.001; DD: *F*_(1, 68)_ = 8.33, *p* < 0.01; VA: *F*_(1, 68)_ = 47.09, *p* < 0.001]. The interaction effects were not significant (*p*s > 0.5), suggesting that the redundancy gain (RG), which is defined by the difference in mean reaction times between the single-target and redundant-target conditions, was consistently found for both groups in all the tasks.

**Table 1:** **Mean performance for both groups of participants in each task in Experiment 1**.

		**Mean accuracy**			**Mean reaction time (ms)**		
**Task**	**Group**	**RT**	**ST**	**NT**	**RT**	**ST**	**RG**
CS	High	1.00	1.00	0.89	374.90	421.76	46.86
	Low	1.00	1.00	0.89	396.02	446.95	50.93
DD	High	1.00	1.00	0.99	370.34	393.59	23.25
	Low	1.00	1.00	0.99	391.13	419.69	28.55
VA	High	1.00	1.00	0.99	310.42	373.99	63.57
	Low	1.00	1.00	0.99	338.19	413.03	74.83

“CS,” “DD,” and “VA” are the abbreviations of the color-shape, double-dot, and visual-auditory detection tasks, respectively. “High” and “Low” denote the high-WMC and low-WMC group. “RT,” “ST,” and “NT” represent the redundant-target, single-target, and no-target conditions, respectively. RG is the abbreviation of redundancy gain and is defined as the difference in mean reaction times between the single-target and redundant-target conditions. Note that the mean reaction time of the no-target condition is not shown because any response in this condition is incorrect for a go/no-go version of the redundant-target detection task.

**Figure 2:**
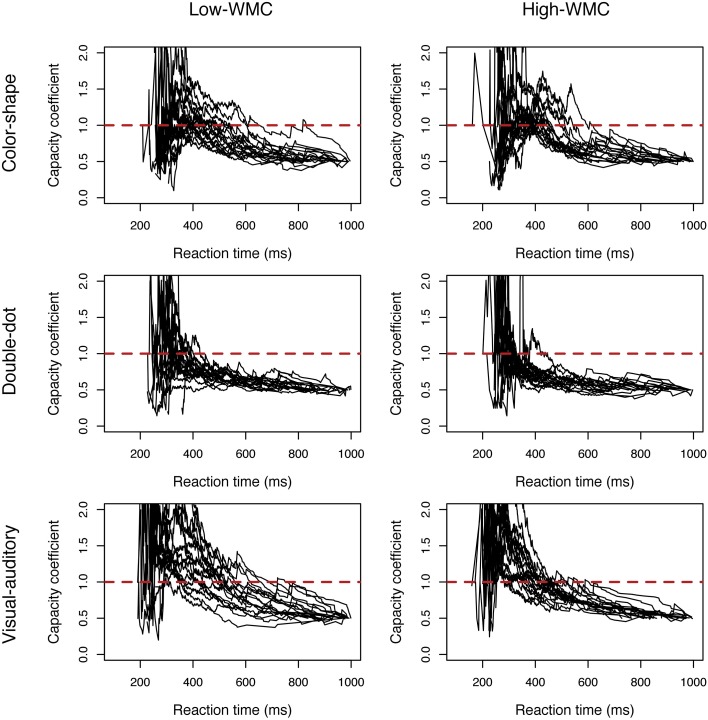
**Plots of the capacity coefficient *C(t)* for the high-WMC and low-WMC groups in each task in Experiment 1**. The red dashed line was the reference line with a value of 1.

*C(t)*s of the three redundant-target detection tasks were computed individually and were plotted by group. Figure 2 showed the results of *C(t)* as a function of reaction time for each group and for each task^3^. From visual inspection, all the results, except for those in the double-dot detection task, showed unlimited-capacity to supercapacity processing. Specifically, in the color-shape detection task, we did not observe any difference in *C(t)* between the high-WMC and low-WMC groups. In this task, both groups of participants had unlimited-capacity (most of the participants had *C(t)* equal to 1) to supercapacity (a few participants had *C(t)* greater than 1 at the faster reaction times). In the double-dot detection task, most participants had limited-capacity processing with *C(t)* less than 1. Lastly, in the visual-auditory detection task, both groups of participants had unlimited-capacity (a few participants had *C(t)* equal to 1) to supercapacity (most of the participants had *C(t)* greater than 1 at the faster reaction times). Specifically, more high-WMC participants had *C(t)* greater than 1 at the faster reaction times than low-WMC partipipants, suggesting that high-WMC participants processed redundant visual and auditory information more efficiently.

To verify these observations, we adopted a non-parametric bootstrapping method to simulate 1000 samples for each condition and to construct the 95% confidence interval for *C(t)* individually (Van Zandt, 2000). If the 95% confidence interval for *C(t)* exceeds 1 at some times *t*, we conclude that the participant adopts supercapacity processing to process multiple signals. If the 95% confidence interval for *C(t)* includes 1 for all times *t*, we conclude that the participant adopts unlimited-capacity processing. Otherwise, we conclude that the participant adopts limited-capacity processing. Table 2 presents the classification results of the inferences based on the simulated data for each group in each task^4^.

**Table 2:** **The WLC classification results of the inferences based on the simulated data for both groups in each task**.

**Task**	**Group**	**Supercapacity**	**Unlimited-capacity**	**Limited-capacity**
CS	High	3	9	6
	Low	3	8	7
DD	High	0	0	18
	Low	0	0	18
VA	High	14	4	0
	Low	9	7	2

“CS,” “DD,” and “VA” are the abbreviations of the color-shape, double-dot, and visual-auditory detection tasks, respectively. “High” and “Low” denote the high-WMC and low-WMC group. The table shows the number of participants who were classified as supercapacity, unlimited-capacity, or limited-capacity for both groups in each task.

Based on the classification results, we then did two levels of analyses. First, we computed the odds ratios between the supercapacity/limited-capacity of the high-WMC group and the supercapacity/limited-capacity of the low-WMC group in different tasks. If the odds ratio equals 1, it suggests that high-WMC and low-WMC groups are classified into different WLC categories similarly, and that they have similar WLC in processing multiple signals. Otherwise, we can conclude that they have different WLC in processing multiple signals. Results showed that the odds ratio in the color-shape detection task was 1.17, suggesting that two groups of participants did not differ from each other in their WLC. In the double-dot detection task, the corrected odds ratio was 1^5^, suggesting that both the high-WMC and low-WMC groups processed multiple signals with limited capacity. In the visual-auditory detection task, the odds ratio was 7.63, suggesting that more high-WMC participants adopted supercapacity processing than low-WMC group participants did.

Second, we fitted the classification data of each task with a multinomial loglinear model which can describe the log expected frequency of each WLC category of different groups (Agresti, 1996). The model consists of a log equation with separate parameters for each WLC category of different groups. We chose the limited-capacity category of the low-WMC group as the baseline category for dummy coding. The intercept describes the log expected frequency of being classified into the baseline category and the estimated parameters for the other category describe the log expected frequency of being classified into the other WLC categories. The Wald test was conducted to examine whether each estimated parameter was significantly different from the frequency of the baseline category. The estimated proportion of being classified into one category and the baseline category can be computed. Results showed that in the color-shape detection task, all the estimated parameters were not significant (*p*s > 0.2), suggesting that the frequencies of being classified into different WLC categories for the high-WMC and low-WMC groups were comparable. That is, both groups had similar WLC in processing multiple signals. In the double-dot detection task, due to all participants being classified as limited-capacity, no further analysis was required. In the visual-auditory detection task, the estimated parameter of the high-WMC group being classified into the supercapacity category was significant [χ^2^_1_ = 6.63, *p* < 0.05], and the estimated proportion between this category and the baseline category was 7. In addition, the estimated parameter of the low-WMC group being classified into the supercapacity category was marginal significant [χ^2^_1_ = 3.7, *p* = 0.054], and the estimated proportion between this category and the baseline category was 4.5. Although for both groups, there were more participants classified into supercapacity category compared to the baseline category, the estimated proportion between the frequency of supercapacity category and that of the baseline category was larger for the high-WMC group than for the low-WMC group, verifying that high-WMC group had larger WLC than the low-WMC group in processing redundant visual and auditory signals.

These results suggested that performance on the OSPAN task can predict the capacity of processing redundant information from different modules; however, WMC cannot predict the capacity for processing redundant featural information of an object and the capacity for processing visual information from two spatial locations.

## Experiment 2

The results of Experiment 1 showed that high-WMC and low-WMC participants differed in their WLC when performing a visual-auditory detection task, but not in the other tasks. However, there were a few limitations in Experiment 1. For example, the results drawn from the non-parametric approach (SFT) can only provide a discrete distinction between the high-WMC and low-WMC groups. We were curious about whether there is a linear relationship between WLC and WMC. Second, with the SFT, we only analyzed the correct reaction times; thus, the incorrect responses were not taken into consideration. Third, we observed a potential response bias in the color-shape detection task in Experiment 1; however, we did not collect reaction time data for the no-go response. Therefore, Experiment 2 was motivated to use a yes/no version of redundant-target detection task and adopt a parametric approach LBA (Brown and Heathcote, 2008) to estimate WLC, the LBA-based capacity, for each participant. With this approach, we incorporated both correct and incorrect reaction times and both target-present and target-absent trials into analyses. The estimated LBA-based capacity can be used to correlate with WMC to test whether there is a linear relationship between WMC and WLC. We aimed to provide converging evidence to support the relationship between WLC and WMC found in Experiment 1.

### Method

#### Participants

Participants included 131 undergraduates at National Cheng Kung University who had not participated in the first experiment. Three participants were not considered in this study because they could not participate in the OSPAN task. There were 53 males and 75 females with an average age of 19 and a standard deviation of 1.33. All the participants had normal or corrected-to-normal vision and hearing. They signed a written informed consent prior to the experiment and received NTD 120 per hour for their participation.

#### Stimuli, design, and procedure

The stimuli, design, and procedure were the same as those used in Experiment 1, except that a yes/no response was required. We adopted a yes/no task instead of a go/no-go task because we needed to collect the reaction times of the no-target condition to estimate the drift rates (rates of the information accumulation) and estimate the parametric measure of WLC (see details in the following **Data analysis** Section). Participants were instructed to press the “/” button when *either* or *both* target features were detected and press the “z” button when *neither* target feature was detected.

#### Data analysis

To estimate the parametric measure of WLC, we adopted the LBA model to analyze the reaction time data of the redundant-target detection tasks. Take the color-shape detection task for an example. Two target features, color (C) and shape (S), require four independent, parallel accumulators that collect evidence: (1) target color is present (i.e., green), (2) target color is absent (i.e., cyan), (3) target shape is present (i.e., X), and (4) target shape is absent (i.e., O). We denoted these accumulators C, ~C, S, and ~S, respectively. Each accumulator collects evidence from a starting point, which is uniformly distributed and ranges from 0 to *A*. A decision is made when the amount of accumulated evidence collected by one of the accumulators reaches the threshold *b*. The information accumulation rate (drift rate) of an accumulator is drawn from a normal distribution with a mean of ν and a standard deviation of *s*. The reaction time can be separated into two components: (1) decision time: the time taken for an accumulator to reach the threshold, and (2) non-decision time (*t_0_*), also called base time, i.e., the time taken for sensory preparation and motor execution. There are a total of five parameters used to describe an accumulator: θ = (b, A, ν, s, t_0_).

In the redundant-target detection task, participants were required to make a yes/no response. A “*YES*” response, indicating that either or both target features are present, is made if either C or S reaches the threshold while ~C, ~S, or both have not reached the threshold. Hence, the overall likelihood of a positive response at time *t* is the sum of the likelihoods of the two events (i.e., C reaches the threshold and S has not, and vice versa.):

(2) $$\begin{array}{l} L(YES,\;t)=\left[1-F_{\sim C}(t)\cdot F_{\sim S}(t)\right]\cdot \\ \;\left[f_{C}(t)\cdot S_{S}(t)+f_{S}(t)\cdot S_{C}(t)\right] \end{array}$$

where *S_i_(t)*, *f_i_(t)*, and *F_i_(t)* represent the survivor function, probability density function, and the cumulative distribution function of the accumulator *i* at time *t*, respectively. A “*NO*” response (neither the target color nor the target shape is present) is made if both ~C and ~S reach the threshold and both C and S have not reached the threshold. Hence, the overall likelihood of a negative response is the sum of the likelihood of the two events (i.e., ~C reaches threshold after ~S reaches the threshold, and vice versa):

(3) $$\begin{array}{l} L(NO,\;t)\;=\;S_{C}(t)\;\cdot \;S_{S}(t)\cdot \\ \;\left[f_{\sim C}(t)\;\cdot \;F_{\sim S}(t)+f_{\sim S}(t)\;\cdot \;F_{\sim C}(t)\right] \end{array}$$

Given a set of parameters for each condition, Equations (2) and (3) were used to evaluate the likelihood of all the correct and incorrect reaction time data. We adopted an optimization algorithm to find a set of parameters that maximized the likelihood separately for each participant. In accordance with Eidels et al. (2010), a total of eleven free parameters were used (i.e., *A, b_T_, b_NT_, t_0RT_, t_0ST_, t_0NT_, v_RT_, v_ST_, v_NT_, v*_~*T*_, *v*_~*NT*_). Because the stimulus encoding of base time may decrease with two targets versus one target due to perceptual factors, we estimated separate base time parameters of *t_0RT_, t_0ST_*, and *t_0NT_* for the redundant-target, single-target, and no-target conditions, respectively. Due to the unequal number of trials between target-present (i.e., redundant-target and single-target condition) and target-absent conditions (i.e., no-target condition), participants might be biased toward making a positive response. Therefore, we estimated separate threshold parameters *b_T_* and *b_NT_* for the target-present and target-absent conditions, respectively. We estimated a single value *A* for the starting point across all responses and conditions. The standard deviation of the drift rate (*s*) was fixed at 1 in the double-dot and visual-auditory detection tasks and at 0.25 in the color-shape detection task in order to obtain the best fit for our models (see Eidels et al., 2010). We assumed five free drift rate parameters, although there could be up to 16. These five parameters were three drift rate parameters when the targets were present (*v_RT_, v_ST_, v_NT_)* and two drift rate parameters (*v_~T_*, *v*_~*NT*_) when the targets were absent. The drift rate parameters were summarized in Table 3. We chose only five parameters because we assumed that drift rates were equivalent for processing C and S and for processing ~C and ~S. This assumption may not be true; however, when we incorporated these parameters into further analysis, we can draw the same conclusion even with a general model that possessed a larger Bayesian information criterion (BIC), indicting a worse fitting than the restricted model.

**Table 3:** **The simplified set of five drift rate parameters (right-hand side) used in the LBA model and their corresponding drift rates of all accumulators (left-hand side) in the redundant-target detection task**.

		**Target color**	
		**Present (C)**	**Absent (~C)**
Target shape	Present (S)	*v*_*C*|*CS*_ = *v_RT_*	*v_C|~ CS_* = *v_~T_*
		*v_S|CS_* = *v_RT_*	*v_S|~CS_* = *v_ST_*
		*v_~C|CS_* = *v_NT_*	*v_~C|~CS_* = *v_~NT_*
		*v_~S|CS_* = *v_NT_*	*v_~S|~CS_* = *v_NT_*
	Absent (~S)	*v_C|C~S_* = *v_ST_*	*v_C|~C~S_* =*v_~T_*
		*v_S|C~S_* = *v_~T_*	*v_S|~C~S_* = *v_~T_*
		*v_~C|C~S_* = *v_NT_*	*v_~C|~C~S_* = *v_~NT_*
		*v_~S|C~S_* = *v_~NT_*	*v_~S|~C~S_* = *v_~NT_*

Subscripts for the simplified set of five drift rates are described in the **Data analysis** Section of Experiment 2. Subscripts for the full set of sixteen drift rate parameters denote the drift rate for a specific accumulator given any of the four test trials. For instance, v_C|CS_ represents the drift rate for the accumulator C when both the target color and target shape are present and is mapped to the drift rate for the redundant-target accumulator v_RT_.

We used the relative difference between *v_RT_* and *v_ST_* as a parametric measure of the WLC. The LBA-based capacity can be expressed as follows:

(4) $$v_{diff}=\nu_{RT}-\nu_{ST}.$$

If *v_RT_* = *v_ST_* then unlimited-capacity processing is suggested. If the drift difference is greater or less than 0, a supercapacity processing (when *v_RT_* > *v_ST_*) or limited-capacity processing (when *v_RT_* < *v_ST_*) is suggested.

#### Result

As in Experiment 1, we estimated the participants' WMC by using the data of the OSPAN task. Ten participants' data were excluded from further analysis because their processing accuracy was below 0.7. Another eleven participant' data were excluded as well because they had relatively slow mean reaction times or low accuracies in the no-target condition when they performed the redundant-target detection tasks. Under these criteria, the mean processing accuracy was 0.86 with a standard deviation of 0.07. The mean recall score was 35.75 with a standard deviation of 10.20. The high-WMC group included the participants with the top 30% of recall scores (*M* = 46.86, *SD* = 5.42, *N* = 36), whereas the low-WMC group included the participants with the bottom 30% of recall scores (*M* = 24.69, *SD* = 5.38, *N* = 36). The difference in recall scores between the high-WMC and low-WMC groups was significant [*t*_(70)_ = 17.41, *p* < 0.0001].

We then excluded the trials with reaction times less than 150 ms in the redundant-target detection tasks for further analyses. The mean performance of the redundant-target detection tasks for each group was summarized in Table 4. Accuracies were very high across conditions for both groups of participants except for the no-target conditions of the color-shape detection task (High: 0.88; Low: 0.89), suggesting a potential response bias in detecting color and/or shape. We will limit the remainder of our analyses to the reaction time. A two-way (high-WMC/low-WMC group × redundant-target/single-target condition) ANOVA was conducted to analyze the accuracy and correct reaction time data of the three tasks. For accuracy data, there were significant main effects of condition [CS: *F*_(1, 140)_ = 148.09, *p* < 0.001; DD: *F*_(1, 140)_ = 16.77, *p* < 0.001; VA: *F*_(1, 140)_ = 187.81, *p* < 0.001], showing lower accuracy in the no-target conditions than in the other two conditions. These results were different from what we found in Experiment 1 where accuracy in most conditions reached the ceiling. For reaction time data, there were significant main effects of group in the color-shape and double-dot detection task [CS: *F*_(1, 140)_ = 12.76, *p* < 0.001; DD: *F*_(1, 140)_ = 5.14, *p* < 0.05; VA: *F*_(1, 140)_ = 0.82, *p* = 0.37] and condition in all the tasks [CS: *F*_(1, 140)_ = 40.42, *p* < 0.001; DD: *F*_(1, 140)_ = 6.98, *p* < 0.01; VA: *F*_(1, 140)_ = 58.05, *p* < 0.001]. The interaction effects were not significant in all the tasks for both groups (*p*s > 0.2), suggesting that the RG was consistently found for both groups in all the tasks.

**Table 4:** **Mean performance for both groups of participants in each task in Experiment 2**.

		**Mean accuracy**			**Mean reaction time (ms)**			
**Task**	**Group**	**RT**	**ST**	**NT**	**RT**	**ST**	**NT**	**RG**
CS	High	1.00	0.97	0.88	346.72	390.01	454.67	43.29
	Low	1.00	0.98	0.89	369.78	418.83	491.96	49.05
DD	High	0.99	0.98	0.93	400.13	420.46	574.43	20.33
	Low	0.99	0.98	0.93	417.35	440.78	595.11	23.43
VA	High	1.00	0.97	0.93	323.27	382.87	539.70	59.60
	Low	1.00	0.97	0.91	329.31	391.29	556.98	61.99

“CS,” “DD,” and “VA” are the abbreviations of the color-shape, double-dot, and visual-auditory detection tasks, respectively. “High” and “Low” denote the high-WMC and low-WMC groups. “RT,” “ST,” and “NT” represent the redundant-target, single-target, and no-target conditions, respectively. RG is the abbreviation of redundancy gain and is defined as the difference in mean reaction times between the single-target and redundant-target conditions.

**Figure 3:**
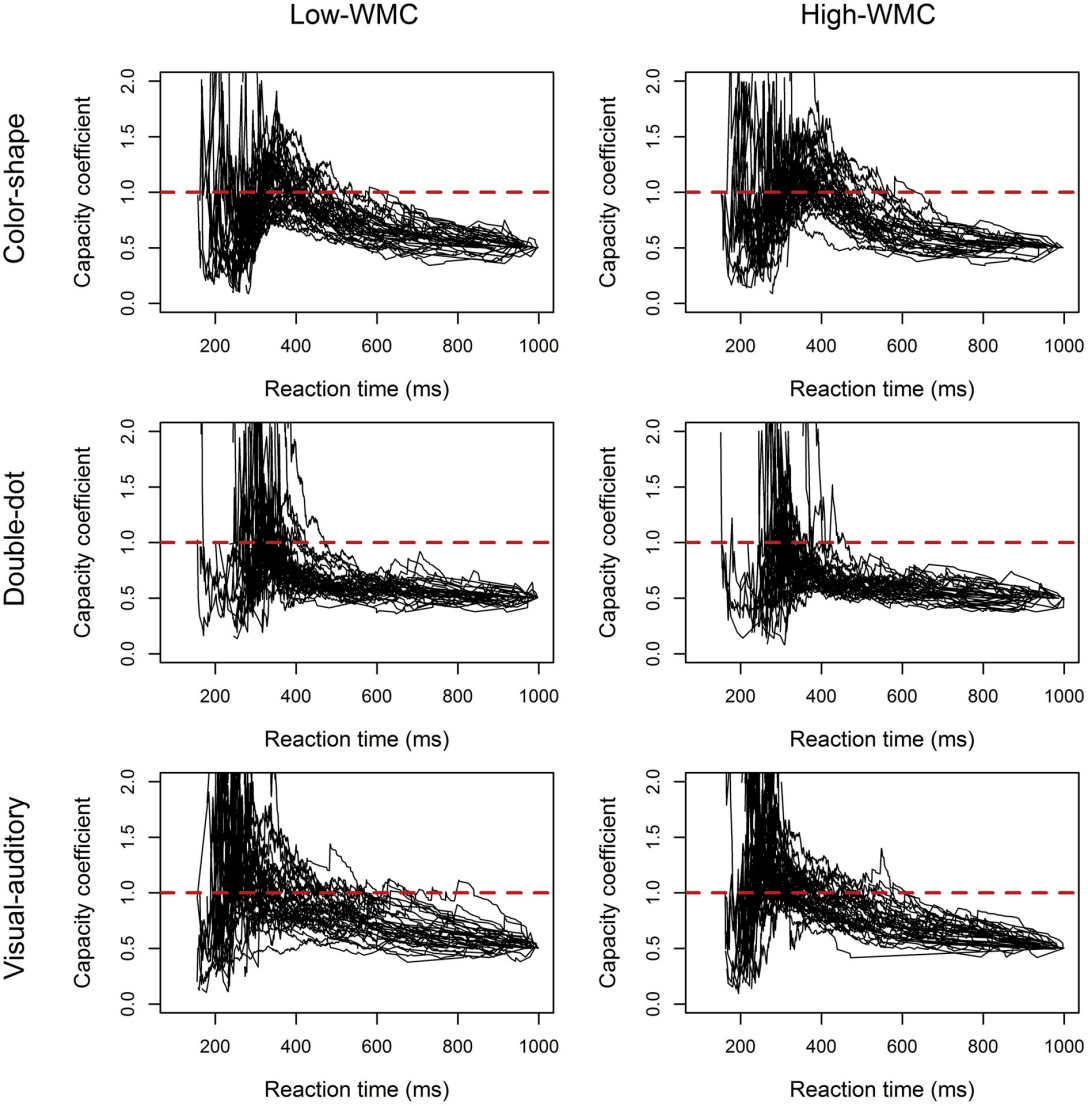
**Plots of the capacity coefficient *C(t)* for the high-WMC and low-WMC groups in each task in Experiment 2**. The red dashed line was the reference line with a value of 1.

*C(t)*s of the three redundant-target detection tasks were computed individually and were plotted by group. Figure 3 showed the results of *C(t)* as a function of reaction time for both groups in each task. The results in Experiment 2 were comparable to those in Experiment 1. In the color-shape and double-dot detection task, no difference in *C(t)* between the high-WMC and low-WMC groups was observed. Both groups of participants had unlimited-capacity in processing color and shape with *C(t)* equal to 1 for all times *t*; however, we found a few participants had *C(t)* greater than 1 at the faster RTs. In the double-dot detection task, most participants had limited-capacity processing with *C(t)* less than 1. Finally, in the visual-auditory detection task, both groups of participants had *C(t)* greater than 1 at the faster RTs, suggesting supercapacity processing. In addition, more high-WMC participants showed this pattern than low-WMC participants did, suggesting that high-WMC participants processed redundant visual and auditory information more efficiently. The results of Experiment 1 can be generalized to a yes/no task.

To verify these observations, we adopted the non-parametric bootstrapping method as Experiment 1 to construct the 95% confidence interval for *C(t)* of all the tasks and for each participant. Table 5 presents the classification results of the inferences based on the simulated data for both groups in each task.

**Table 5:** **The WLC classification results of the inferences based on the simulated data for both groups in each task**.

**Task**	**Group**	**Supercapacity**	**Unlimited-capacity**	**Limited-capacity**
CS	High	2	26	7
	Low	2	24	10
DD	High	2	1	33
	Low	2	2	32
VA	High	13	21	1
	Low	11	21	3

“CS,” “DD,” and “VA” are the abbreviations of the color-shape, double-dot, and visual-auditory detection tasks, respectively. ”High” and “Low” denote the high-WMC and low-WMC group. The table shows the number of participants who were classified as supercapacity, unlimited-capacity, or limited-capacity for both groups in each task.

We then computed the odds ratios between the supercapacity/limited-capacity of the high-WMC and the supercapacity/limited-capacity of the low-WMC group in the three tasks. Results showed that the odds ratios were 1.42 in the color-shape detection task and 0.97 in the double-dot detection task, suggesting that the two groups were classified into different WLC categories similarly. In the visual-auditory detection task, the odds ratio was 3.55, suggesting that more participants adopted supercapacity processing in the high-WMC group than in the low-WMC group.

The results analyzed with the multinomial loglinear model also supported our observations. Results showed that in the color-shape detection task, the estimated parameters of the high-WMC group being classified into the supercapacity category and unlimited-capacity category were significant (supercapacity: χ^2^_1_ = 4.32, *p* < 0.05; unlimited-capacity: χ^2^_1_ = 6.59, *p* < 0.05), and the estimated proportions between these categories and the baseline category were 0.2 and 2.6. Also, the estimated parameters of the low-WMC group showed a similar pattern of results (supercapacity: χ^2^_1_ = 4.32, *p* < 0.05; unlimited-capacity: χ^2^_1_ = 5.41, *p* < 0.05), and the estimated proportions between these categories and the baseline category were 0.2 and 2.4. These results suggested that the two groups were classified into different WLC categories similarly. In the double-dot detection task, the results of the estimated parameters were significant for both the high-WMC (supercapacity: χ^2^_1_ = 14.47, *p* < 0.001; unlimited-capacity: χ^2^_1_ = 11.65, *p* < 0.001) and low-WMC groups (supercapacity: χ^2^_1_ = 14.47, *p* < 0.001; unlimited-capacity: χ^2^_1_ = 14.47, *p* < 0.001). The estimated proportions between high and supercapacity, high and unlimited-capacity, low and supercapacity, and low and unlimited-capacity categories and the baseline category were 0.06, 0.03, 0.06, and 0.06, respectively, suggesting more participants were classified into limited-capacity category for both groups. In the visual-auditory task, the estimated parameters were significant for both the high-WMC (supercapacity: χ^2^_1_ = 5.24, *p* < 0.05; unlimited-capacity: χ^2^_1_ = 9.94, *p* < 0.005) and low-WMC groups (supercapacity: χ^2^_1_ = 3.98, *p* < 0.05; unlimited-capacity: χ^2^_1_ = 9.94, *p* < 0.005). The estimated proportions between high and supercapacity, high and unlimited-capacity, low and supercapacity, and low and unlimited-capacity categories and the baseline category were 4.33, 7, 3.67, and 7, respectively. The estimated proportion between the supercapacity category and the baseline category was larger for the high-WMC group than for the low-WMC group, verifying that the high-WMC group had larger WLC than the low-WMC group in processing redundant visual and auditory signals.

However, comparing the results between the two experiments, we found that fewer participants were classified into supercpacity category in Experiment 2 than in Experiment 1. This discrepancy may be due to the nature of the tasks used in the two experiments (go/no-go vs. yes/no tasks). It is worthy to note that our findings were consistent with the previous research (Blurton et al., 2014), in which the race-model inequality was easily violated in a go/no-go task compared to a forced-choice task.

**Figure 4:**
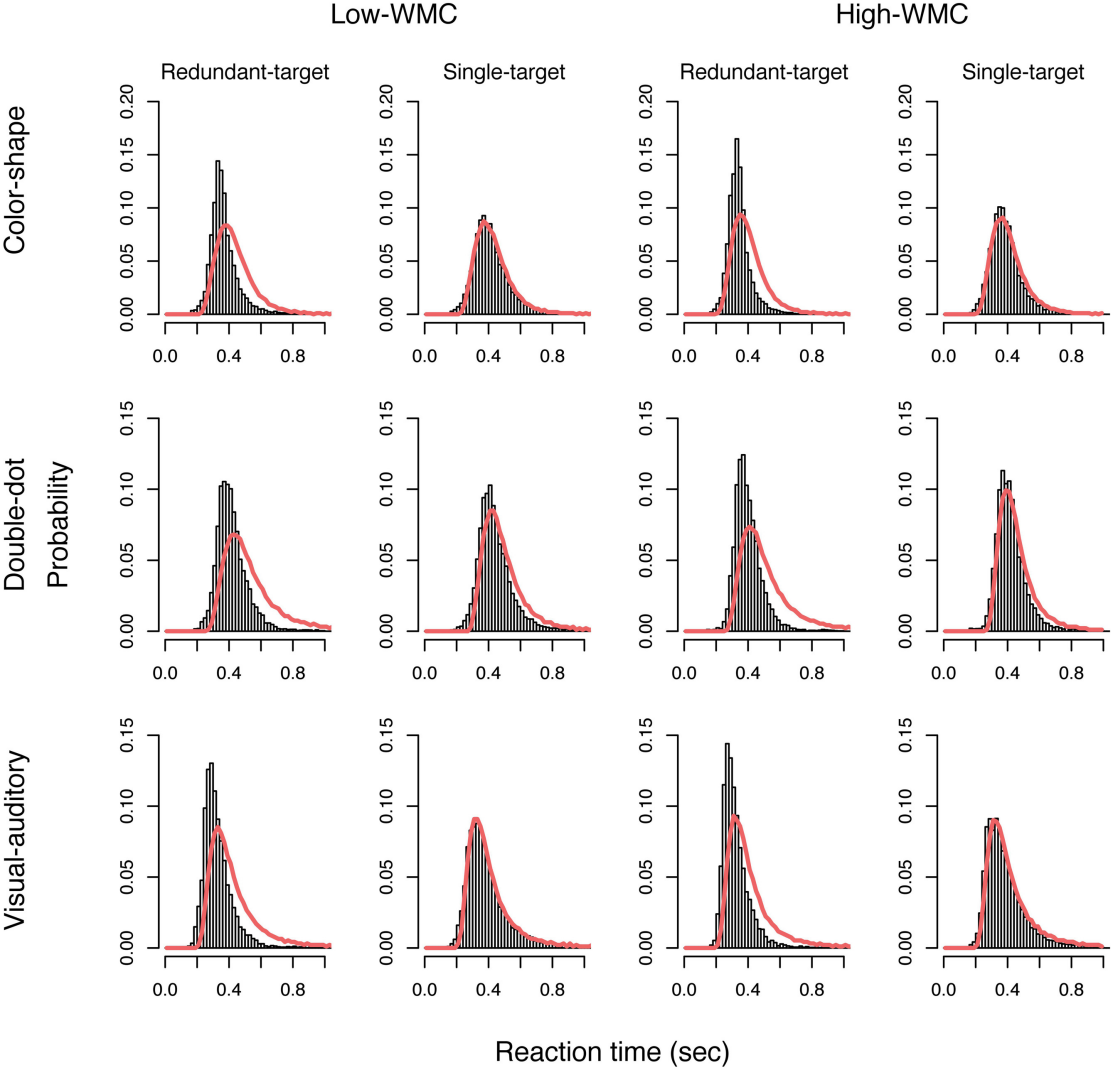
**Plots of the predicted density functions (the red solid line) on top of the empirical histograms of the redundant-target and single-target conditions for each group in each task**.

Next, we used the LBA model to analyze the reaction time data and estimated a set of parameters that maximized the likelihood function described in the **Method** Section for each participant. Table 6 presented the average of 11 estimated parameters for both groups in different tasks. We then used the average of the estimated parameters to simulate data and plotted the model predictions based on the simulated data on top of the empirical histogram (see Figure 4). Results showed that the LBA model fitted the participants' reaction time data because the predicted density from the model can capture the empirical density successfully.

**Table 6:** **The average values of eleven estimated parameters and the LBA-based capacity (*v_Diff_*) for both groups of participants in each task**.

		**Estimated parameters**											
**Task**	**Group**	***A***	***t_**0**_*RT**	***t_**0**_ST***	***t_**0**_NT***	***b_**T**_***	***b_**NT**_***	***v_**RT**_***	***v_**ST**_***	***v_**NT**_***	***v_~**T**_***	***v_~**NT**_***	***v_**Diff**_***
CS	High	165.64	54.81	51.45	26.79	439.67	532.07	1.08	1.06	0.42	0.67	1.23	0.02
	Low	199.93	60.86	63.15	23.59	476.50	609.03	1.07	1.08	0.38	0.62	1.27	−0.02
DD	High	477.73	156.91	175.91	159.98	1080.78	1031.60	2.67	3.28	−0.85	−0.82	2.47	−0.61
	Low	516.01	140.98	164.25	136.27	1214.55	1104.19	2.72	3.41	−0.29	−0.46	2.44	−0.69
VA	High	269.24	104.98	101.26	89.21	841.62	948.63	2.63	2.52	−0.38	0.09	2.39	0.11
	Low	296.69	90.34	93.20	50.35	930.58	1060.69	2.64	2.81	−0.51	0.28	2.40	−0.18

“CS,” “DD,” and “VA” are the abbreviations of the color-shape, double-dot, and visual-auditory detection tasks, respectively. “High” and “Low” denote the high-WMC and low-WMC groups.

**Figure 5:**
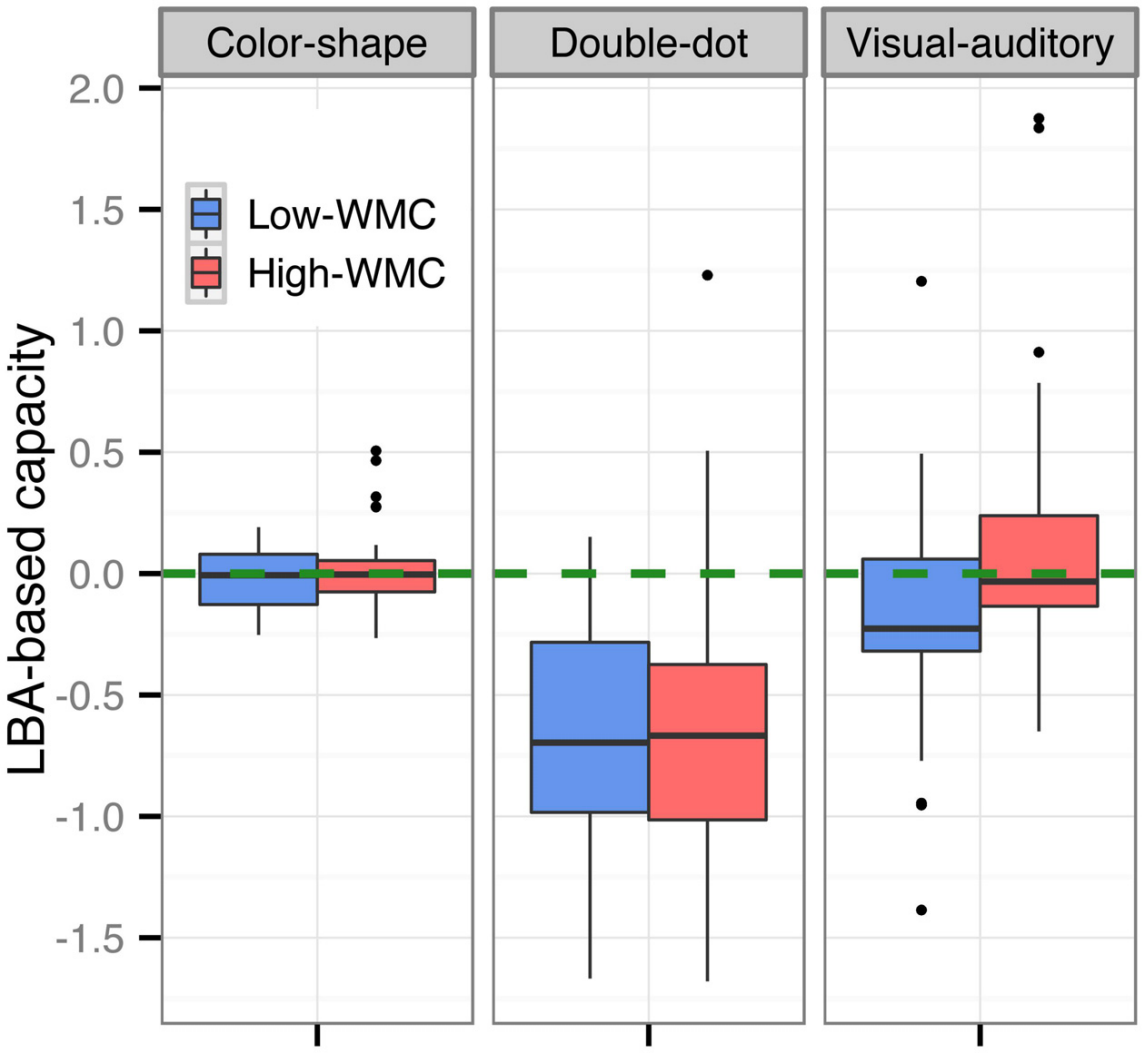
**Boxplots of the LBA-based capacity of the high-WMC and low-WMC groups in each task**. The green dashed line was the reference line with a value of 0. The difference in the LBA-based capacity between the high-WMC and low-WMC groups reached the significance level only in the visual-auditory detection task, but not in the other two tasks.

**Figure 6:**
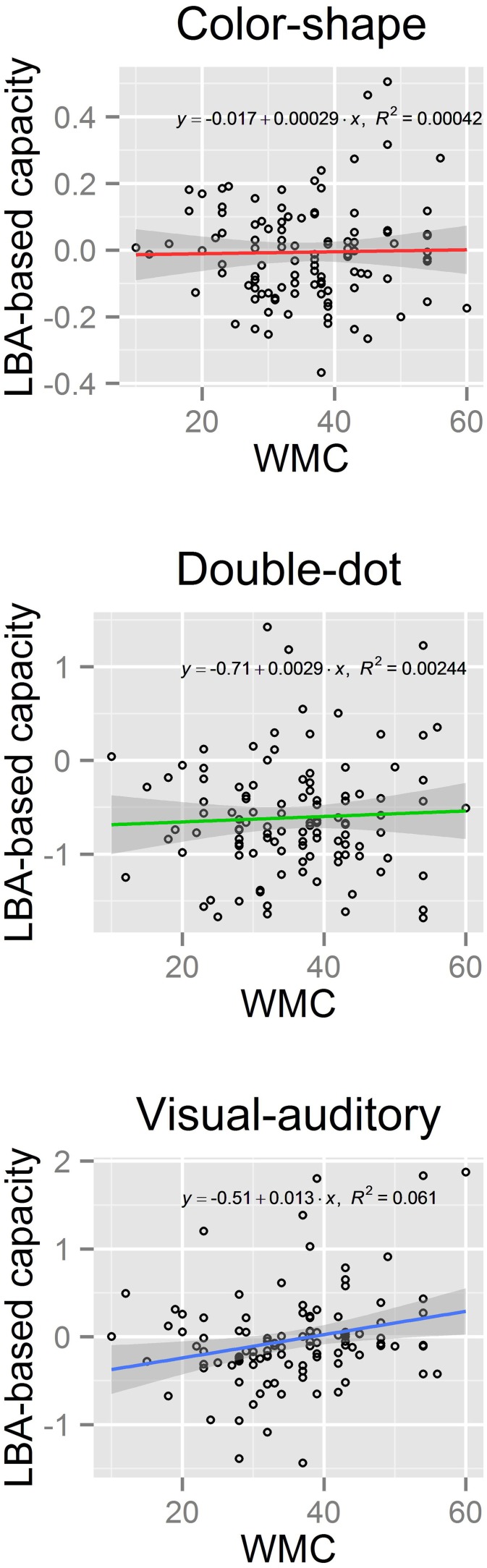
**Scatter plots of the LBA-based capacity and the WMC scores with a regression line (solid colored line) and its 95% confidence interval (band-shaped gray area) of each task**. A significant positive correlation between WMC and WLC was found in the visual-auditory detection task, but not in the other two tasks.

We then computed the LBA-based capacity for both groups in each task (see Figure 5). Results showed a significant difference in the LBA-based capacity between the high-WMC and low-WMC groups in the visual-auditory detection task [*t*_(70)_ = 2.36, *p* < 0.05]; however, this difference was not observed in the color-shape detection task (*p* = 0.35) and in the double-dot detection task (*p* = 0.55). Finally, we computed the Pearson's product-moment correlation (*r)* between the recall scores and the LBA-based capacity. A significant positive correlation between the WMC and WLC was found in the visual-auditory detection [*r* = 0.25, *p* < 0.01, 95% CI = (0.06, 0.41)], whereas the correlations in the color-shape detection task [*r* = 0.02, *p* = 0.83, 95% CI = (−0.17, 0.21)] and double-dot detection task [*r* = 0.05, *p* = 0.61, 95% CI = (−0.14, 0.24)] did not reach the significance level (see Figure 6). These results provided converging evidence showing that participants high in WMC had larger WLC only in the visual-auditory detection task.

## Discussion

We examined the relationship between WMC and WLC, and tested whether the two capacity measures assessed a unitary, central capacity of information processing. We used an OSPAN task to assess WMC and three different redundant-target detection tasks to assess WLC. We conducted an extreme-group approach to split the participants' data according to their WMCs and compared them to their WLCs in both experiments, and computed the Pearson's product-moment correlation to verify the linear relationship between the two capacity measures in Experiment 2. WLC was estimated with the reaction time data of the redundant-target detection tasks both non-parametrically (SFT in Experiments 1 and 2) and parametrically (LBA in Experiment 2). The results from the two experiments showed that participants high in WMC had a larger perceptual processing capacity in detecting multiple signals from different modalities (the visual-auditory detection task); this difference was eliminated when multiple signals came from different object features (the color-shape detection task) and from different spatial locations (the double-dot detection task). These results suggested that the individual differences in WMC can predict the ability to process multiple sources of information in a certain perceptual task and shed light on the functioning of the central executive system of working memory in multiple-signal processing. Further implications on the nature of a working memory system will be discussed.

In the model of working memory (Baddeley and Hitch, 1974), central executive system plays an important role in maintaining, updating, operating, and integrating information between percepts and the two subsystems, which store visuospatial and phonological information, respectively. In previous research on working memory, measures of WMC are strongly correlated to performance in various complex cognitive tasks, such as reading comprehension (McVay and Kane, 2012), logical reasoning (Oberauer et al., 2007), problem solving (Hoffman and Schraw, 2009), and creative thinking (Dietrich, 2004). In addition, differences in WMC can account for variation in individuals' general intelligence quotient (IQ) (Engle et al., 1999; Kane and Engle, 2002; Conway et al., 2003). Previous researchers suggest that WMC reflects the efficiency of the central executive system in maintaining and processing information (see Barrett et al., 2004 for a review), most notably the ability to selectively maintain task-relevant information (Redick et al., 2007; Lecerf and Roulin, 2009; Minamoto et al., 2010). WMC also reflects individual differences in the ability to focus and maintain attention in binding and integrating multiple sources of information (Barrett et al., 2004), particularly when a salient distractor is likely to capture attention; this ability may decrease with age (Palladino and Beni, 1999). Recently, a number of neuroimaging studies have demonstrated the role of the prefrontal cortex in executive function (e.g., Miller and Cohen, 2001; Kane and Engle, 2002).

On the other hand, WLC measures the variation of the processing efficiency of an individual channel as a function of workload (Townsend and Ashby, 1978; Townsend and Nozawa, 1995; Wenger and Gibson, 2004; Townsend and Eidels, 2011). In previous research, WLC has been assessed with a redundant-target detection task (see Townsend and Nozawa, 1995 for a review) in various perceptual domains, such as simple detection (Townsend and Eidels, 2011), visual search (Fifić et al., 2008), memory search (Townsend and Fifić, 2004), face perception (Fifić et al., 2008), categorization (Fifić et al., 2010), multisensory perception (Altieri and Townsend, 2011), and change detection (Yang, 2011; Yang et al., 2011, 2013). WLC is likely to constrain the order of multiple-signal processing. For example, a coactive system is usually of supercapacity (Wenger and Townsend, 2001); an independent parallel system is found to be of unlimited-capacity (Houpt and Townsend, 2012); a standard serial model is of limited-capacity (Townsend and Ashby, 1983). In addition, according to Eidels et al. (2011), multiple processes may interact with each other when a parallel system is of supercapacity or limited-capacity processing. Therefore, it is reasonable to speculate that when participants have a system of larger processing capacity, especially supercapacity, they can process redundant information more efficiently and the multiple processes can be completed in a coactive fashion, or that there would be facilitatory between-channel crosstalk during information accumulation such that the participants can optimize the use of multiple signals in perceptual decision making. In contrast, when participants have a system of limited-capacity processing, they are limited in processing multiple signals such that multiple processes may be completed in a serial fashion. Limited-capacity processing may also indicate that there is an inhibitory interaction during information accumulation, such that processing one channel of information can inhibit the other process, leading to slower individual-channel processing.

Instead of aggregating all the participants' data to do group analysis, a few recent studies inferred individuals' information processing characteristics by examining their reaction time data, focusing mostly on the individual differences in their processing strategies and processing capacity. For example, Yang et al. (2011) found individual differences in processing strategies when participants were required to detect a luminance change and an orientation change of a Gabor patch, and the relative decision difficulty between the two feature-changes were not controlled. One group of participants adopted serial self-terminating processing with limited capacity, and the other group adopted coactive processing with supercapacity. In Yang's (2011) study, when relative saliency existed in detecting an orientation change and a frequency change of a Gabor patch, three participants adopted serial self-terminating processing with limited-capacity to unlimited-capacity processing to detect changes, while one participant adopted parallel self-terminating processing with unlimited-capacity processing. Similarly, in a categorization task, Fifić et al. (2010) found that participants used multiple sources of information differently to make a categorization decision. However, these studies did not explain the causes of individual differences in processing strategies. We speculated that limits in the processing capacity might constrain the information processing strategy. These individual variations in processing capacity can be predicted by ones' capacity of executive attentional control of a working memory system in processing information.

Although WMC and WLC were proposed around the same time, no prior studies, except for a recent one conducted by Heathcote et al. (2014), investigated the relationship between the two capacity measures. Theoretically, the two capacity measures assessed some similar characteristics of information processing. Most notably, controlled attention played an important role in a working memory system (Rosen and Engle, 1997; Engle et al., 1999; Barrett et al., 2004; Engle and Kane, 2004) and in multiple-signal processing at perception, such as feature integration (Treisman and Gelade, 1980), goal-derived visual selection (Bargh, 1982), perceptual organization (Mack et al., 1992), and perceptual learning (Shiffrin and Schneider, 1977). Thus, it was reasonable to hypothesize that these two measures may relate to each other to a certain extent.

Heathcote et al. (2014) adopted a mnemonic redundant-target task to measure WLC. Participants were required to respond if either the auditory or visual target was presented two-back in a trial sequence. This task incorporated the test for working memory into the measurement of WLC, which was different from the OSPAN task used in assessing WMC. They also followed the SFT to estimate WLC by comparing the reaction time data between the redundant-target and single-target conditions. Unfortunately, their preliminary results did not show a clear relationship between the measurements of WLC and WMC. They suggested that these two capacity measures did not assess a unitary, central processing capacity. However, the fact that they could not find a significant correlation may be due to the lack of statistical power.

The present study used three perceptual redundant-target detection tasks instead of the mnemonic redundant-target task used by Heathcote et al. (2014) and tested the relationship between WLC and WMC. We found interesting results. First, we found significant differences in the LBA-based capacity between different perceptual tasks [*F*_(2, 210)_ = 47.57, *p* < 0.0001] (see Figure 5), and the results from the non-parametric analyses confirmed this pattern of results (see Figures 2, 3, Tables 2, 5); however, the non-parametric results also showed variations between individuals. Generally, processing capacity was the largest in the visual-auditory task, then in the color-shape detection task, and smallest in the double-dot detection task. These results were consistent with prior research. For example, a number of studies have demonstrated that processing multisensory information was of supercapacity, which was known as an effect of “multisensory integration” (Hugenschmidt et al., 2010; Altieri and Townsend, 2011). One of the best-known studies conducted by Miller (1982) showed that when participants performed a visual-auditory detection task, the race-model inequality (RMI) was violated, suggesting that participants adopted coactive processing with supercapacity in processing multisensory information. Our study found that the LBA-based capacity was greater than or equal to 0 and that most participants had *C(t)* greater than 1 at the faster RTs, indicating supercapacity processing of information from different modalities. On the other hand, Mordkoff and Yantis (1991, 1993) have tested the processing for color and shape of an object. In their studies, the race-model inequality was violated when inter-stimulus contingency existed, while it was not violated when there was no inter-stimulus contingency. In the present study, we did not manipulate the inter-stimulus contingency, and we found that the LBA-based capacity was equal to 0, which was consistent with Mordkoff and Yantis's (1991) findings of unlimited-capacity processing without any manipulation of probability information. Lastly, a few studies have demonstrated limited-capacity processing in double-dot detection. The present study also found that the LBA-based capacity was less than 1 in the double-dot detection task, indicating limited-capacity processing. However, when we looked at the non-parametric results (see Figures 2, 3), we found a few participants had *C(t)* greater than or equal to 1 at the faster RTs, indicating that they may process multiple spatial locations with supercapacity or unlimited-capacity processing. Even though, most participants had *C(t)* less than 1 for all times *t*, indicating limited-capacity processing.

Most interestingly, we found differences in WLC between the high-WMC and low-WMC groups. The differences were only found in the visual-auditory detection task, but not in the other two tasks, and this difference was comparable between the two experiments (see Figures 2, 3, 5). Figure 6 also shows that there was a significant positive correlation between WMC and WLC only when participants performed a visual-auditory detection task. These results suggested that WMC correlated to WLC only when a system needed to integrate multiple signals from two different subsystems (i.e., visuospatial sketchpad and phonological loop) for manipulation, operation, and decision making. This relationship was not observed when a system integrated multiple signals that only required resources from a single subsystem (i.e., the visuospatial sketchpad in the present study). These results indicated that a domain-general resource was required for the controlled attention to integrate and bind multisensory information for decision making. On the other hand, processing redundant information from a single modality required a domain-specific resource that was not necessarily related to WMC. Nonetheless, future studies should examine the individual differences in information processing of a single subsystem, as we did not test the processing of redundant information that originated from a single auditory modality. Individual differences can be discovered by increasing the sample size to increase the statistical power and by testing its generalizability in different experimental contexts.

## Conclusion

We examined the relationship between WMC and WLC. Both the non-parametric and parametric analyses showed that participants high in WMC had larger WLC in processing redundant information from different modalities, suggesting that they processed redundant visual and auditory signals more efficiently and multiple processes were likely to be completed in a coactive fashion. However, the difference was not observed when processing redundant information from a single visual modality. The results highlighted the role of controlled attention in information integration of working memory and multiple-signal processing at perception and further contributed to the understanding of the nature of a working memory system.

## Author contributions

Ju-Chi Yu—Data acquisition, data analysis, and drafting the manuscript. Ting-Yun Chang—Programming, data collection, and data analysis. Cheng-Ta Yang—Conception and design, data interpretation, drafting the manuscript, and final approval.

### Conflict of interest statement

The authors declare that the research was conducted in the absence of any commercial or financial relationships that could be construed as a potential conflict of interest.

## Footnotes

^1^ One might argue that the distance between the two dots was too far, such that participants may adopt a serial processing strategy with reduced WLC. However, in Yang et al. (2014), we used a similar display setting and we found that participants adopted parallel processing when they did not have any prior information about the target location. In addition, in one of our unpublished studies, we used an eye tracker to record the participants' eye movements when they were detecting the double dots and no eye movements were found even when the distance between the two dots was 16 degree.

^2^ CS, DD, and VA are the abbreviations of the color-shape, double-dot, and visual-auditory detection tasks, respectively.

^3^ We thank Dr. James T. Townsend for providing us the guideline to draw the figure of *C(t)*. He suggested re-scaling the figure to emphasize the value of 1 because the inference of processing capacity is made based on the comparison between the value of *C(t)* and the value of 1.

^4^Figure 2 presents the estimated *C(t)* for each group. We do not plot the confidence interval individually in Figure 2 due to information complexity. We summarize the inferences based on the bootstrapping results in Table 2.

^5^ Because the value of some cells is zero, we followed Haldane's correction (Haldane, 1956) to compute the corrected odds ratio by adding 0.5 to each cell.
